# Evaluation of Serum Immunoglobulins (IgG, IgA, IgM) and Circulating Immune Complexes in Oral Precancer and Cancer Patients

**DOI:** 10.1055/s-0041-1725157

**Published:** 2021-03-31

**Authors:** Pooja Madki, Mandya Lakshman Avinash Tejasvi, Geetha Paramkusam, Ruheena Khan, Shilpa J.

**Affiliations:** 1 Department of Oral Medicine and Radiology, SB Patil Dental College and Hospital, Karnataka, India; 2 Department of Oral Medicine and Radiology, Kamineni Institute of Dental Sciences, Narketpally, Telangana, India; 3 Private Practice, Sri Venkateswara Dental Clinic, Hyderabad, Telangana, India; 4 Private Practice, Hyderabad, Telangana, India

**Keywords:** serum immunoglobulin, circulating immune complex, oral precancer, oral cancer patients, oral squamous cell carcinoma

## Abstract

**Objectives** The aim of the present study is to evaluate the role of immunoglobulins (IgA, IgG, and IgM) and circulating immune complexes (CIC) as tumor marker in oral cancer and precancer patients.

**Materials and Methods** The present study was performed on 45 individuals subdivided into three groups, that is, oral precancer, oral cancer and healthy individuals, and levels of immunoglobulins, and CIC was estimated by turbidometry and ELISA method.

**Results** In the present study, the mean serum IgA levels in oral precancer were 161.00 ( ±  118.02) mg/dL, oral cancers were 270.67 ( ±  171.44) mg/dL, and controls were 133.73 ( ±  101.31) mg/dL. Mean serum levels of IgG in oral precancer were 1,430.87 ( ±  316) mg/dL, oral cancers were 1,234.27 ( ±  365.42) mg/dL, and controls were 593.87 ( ±  323.06) mg/dL.

**Conclusion** We found that the levels of serum IgG and IgA were elevated consistently in precancer and cancer group, and Serum IgM levels were increased only in precancer. Also, significant increase in serum CIC levels were seen in oral precancer and cancer group on comparison with control.

## Introduction

Oral cancer is an insidious devastating malignancy and is one of the five leading cancers in India. Among the oral tumors, 90% of them are squamous cell carcinomas (SCC), arise from the mucosal lining. 1 Oral cancer is generally preceded by potentially malignant lesions and conditions for a varying length of time. Early detection of these diseases with the discovery of immunological markers at a clinical, histological, and molecular level has significant reduction in mortality and implementation of multidisciplinary treatment programs, leading to improvement of survivorship and better quality of life. 2

## Materials and Methods

This study includes three main groups with 15 oral premalignant patients, 15 oral cancer patients, and 15 healthy individuals with an age of 20 years and above. Clinically and histopathologically diagnosed oral premalignant and oral cancer patients were included after obtaining an informed consent from the patients. In total, 5 mL of blood was collected by venipuncture using 24-gauge needles, and serum was separated by centrifugation at 2,500 rpm for 15 minutes. The separated supernatant was used for further estimation of serum immunoglobulins and circulating immune complexes.

Serum immunoglobulins were quantified by using a diagnostic kit (Quantia-Ig turbidometric immunoassay for the estimation of immunoglobulin). In principle, the method consists of turbidometric immunoassay for the detection of immunoglobulins in human sera is based on the principle of agglutination reaction. The test specimen is mixed with activation buffer (R1). Anti-human reagent (R2) is then added and allowed to react. Presence of immunoglobulins in the test specimen results in formation of an insoluble complex producing a turbidity, which is measured at wavelength 340 nm. The extent of turbidity corresponds to the concentration of immunoglobulins in the test specimen.

Circulating immune complexes levels were assessed by ELISA method. In total, 100 µL of each calibrator, control, and 1:101 diluted sample to the appropriate wells. Incubate for 30 minutes and wash. Add 100 µL of conjugate to each well. Incubate for 30 minutes and wash and then add 100 µL of substrate to each well. Incubate for 30 minutes and wash. Finally, add 100 µL of stop solution to each well to stop the reaction and measure the absorbance at 450 nm.

## Statistical Analysis

The statistical analysis was performed by using SPSS 19.0 version software. Comparison of groups were done by Kruskal–Wallis ANOVA test and Mann–Whitney U test. A *p* -value <0.005 was considered statistically significant.

## Results

**Fig. 1:**
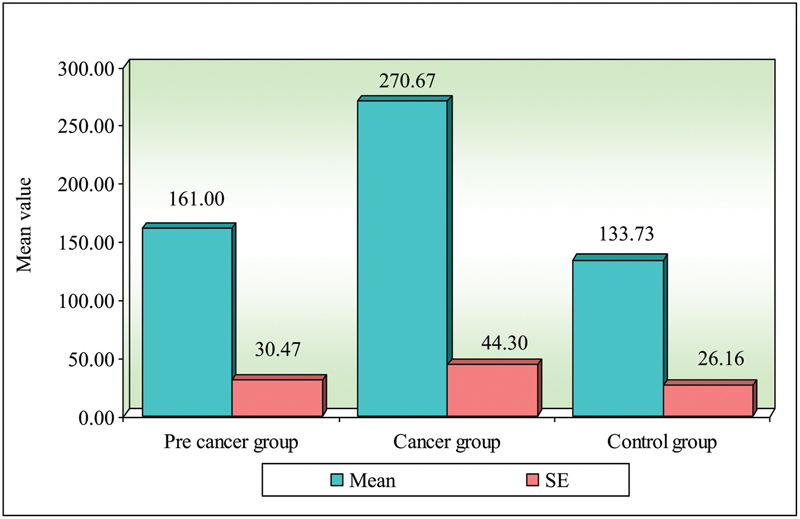
Comparison of three groups (precancer, cancer, and control) with respect to IgA.

In the present study, the mean serum IgA levels in oral precancer were 161.00 ( ±  118.02) mg/dL, oral cancers were 270.67 ( ±  171.44) mg/dL, and controls were 133.73 ( ±  101.31) mg/dL. On comparison of three groups, it was found that serum levels of IgA in oral cancer in comparison with controls were statistically significant when compared with precancers. Mean serum levels of IgG in oral precancer were 1,430.87 ( ±  316) mg/dL, oral cancers were 1,234.27 ( ±  365.42) mg/dL, and controls were 593.87 ( ±  323.06) mg/dL. On comparison of three groups, it was found that comparison between precancer and controls ( *p* < 0.00001). Oral cancers and control (0.0003) were statistically significant, and the mean serum IgM values in oral precancers were 125.60 ( ±  74.92) mg/dL, oral cancers were 59.80 ( ±  41.62) mg/dL, and controls were 62.53 ( ±  28.58) mg/dL. On comparison of three groups was done with respect to serum IgM, it was found that serum IgM level in oral precancer and cancer and oral precancer and controls were statistically significant were as comparison of oral cancer with control was not significant ( Tables 1 2 3 4 , Figs. 1 2 3 4 ).

**Fig. 2:**
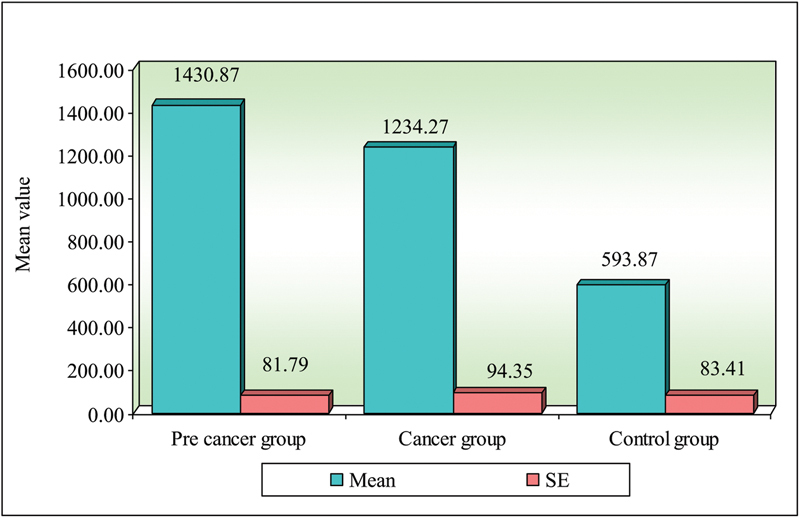
Comparison of three groups (precancer, cancer, and control) with respect to IgG.

**Fig. 3:**
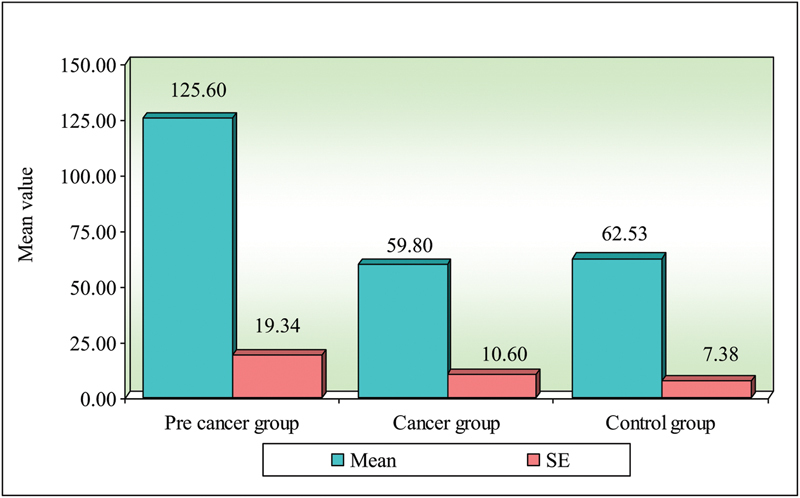
Comparison of three groups (precancer, cancer, and control) with respect to IgM.

**Fig. 4:**
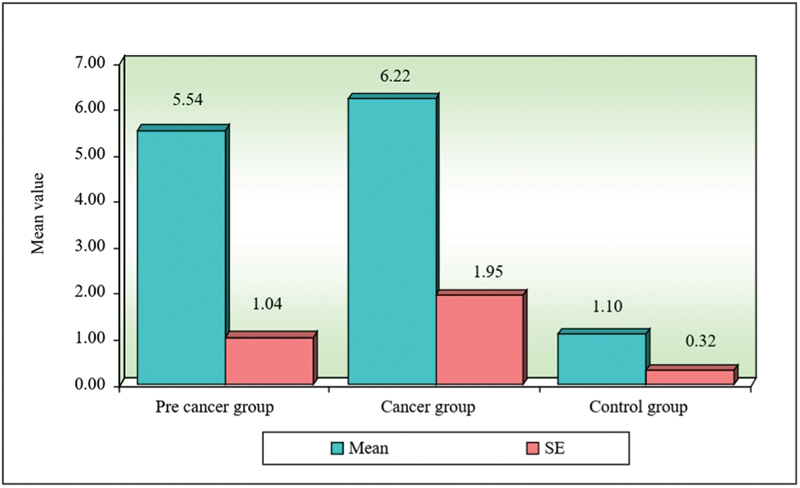
Comparison of three groups (precancer, cancer, and control) with respect to circulating immune complexes.

**Table 1:** Comparison of three groups (precancer, cancer, and control) with respect to IgA scores by Kruskal–Wallis ANOVA test

Groups	Mean	SD	SE	Mean rank
Precancer group	161.00	118.02	30.47	20.90
Cancer group	270.67	171.57	44.30	29.80
Control group	133.73	101.31	26.16	18.30
H-value	6.3340			
*P* -value	0.0420 a			

Abbreviations: SD, standard deviation; SE, standard error.

a *p* < 0.05.

**Table 2:** Comparison of three groups (precancer, cancer, and control) with respect to IgG scores by Kruskal–Wallis ANOVA test

Groups	Mean	SD	SE	Mean rank
Precancer group	1430.87	316.78	81.79	32.70
Cancer group	1234.27	365.42	94.35	26.20
Control group	593.87	323.06	83.41	10.10
H-value	23.5460			
*p* -value	0.00001 a			

Abbreviations: SD, standard deviation; SE, standard error.

a *p* < 0.05.

**Table 3:** Comparison of three groups (precancer, cancer, and control) with respect to IgM scores by Kruskal–Wallis ANOVA test

Groups	Mean	SD	SE	Mean rank
Precancer group	125.60	74.92	19.34	32.03
Cancer group	59.80	41.06	10.60	17.17
Control group	62.53	28.58	7.38	19.80
H-value	10.9580			
*p* -value	0.0040 a			

Abbreviations: SD, standard deviation; SE, standard error.

a *p* < 0.05.

**Table 4:** Comparison of three groups (precancer, cancer, and control) with respect to circulating immune complexes scores by Kruskal–Wallis ANOVA test

Groups	Mean	SD	SE	Mean rank
Precancer group	5.54	4.02	1.04	28.63
Cancer group	6.22	7.55	1.95	23.93
Control group	1.10	1.23	0.32	16.43
H-value	6.6150			
*p* -value	0.0370 a			

Abbreviations: SD, standard deviation; SE, standard error.

a *p* < 0.05.

## Discussion

A tumor marker is a substance present in or produced by host in response to a tumor that can be used to differentiate a tumor from normal tissue or to determine the presence of a tumor based on measurements in blood or secretions. 3 The range of tumor markers in human cancers include enzymes, hormones, polyamines, tumor associated antigens, CIC, lipids, viral markers, oncofetal proteins immunoglobulins, and glycoproteins. 4 Tumor markers in serum, tissue and other body fluids during neoplastic process are of clinical value in the management of patients with various body cancers. Among all the body fluids, blood has been the media of choice for the study of the biochemical markers by the medical community. 5

These markers can be normal endogenous products that are produced at a greater rate in cancer cells. Measurement of these tumor marker is useful in detection and diagnosis of cancers. 6

Serum immunoglobulins are proteins of animal origin with known antibody activity and certain protein related to them by chemical structure and have antigenic specificity. Immunoglobulins are normally found in other body fluids and tissues such as serum, urine, spinal fluid, milk, saliva, tears, lymph nodes, and spleen. 7 They are normally synthesized by the immune system cells to fight against the foreign substances/antigens. 4 These are seen to be produced by plasma cells and lymphocytes at various stages of differentiation. 7

Five distinct classes of immunoglobulin molecules namely IgG, IgA, IgM, IgD, and IgE are recognized in humans. IgM is the class that appears initially when an organism is exposed to an antigen for the first time (primary infection). IgA is the major immunoglobulin class found in mucosal secretions and prevents mucosal infections by agglutinating microbes. 8 IgG is the predominant immunoglobulin in normal serum (70–75%, ∼1,000 mg/dL). 9 Immunoglobulins are the carriers of humoral immunity, and hence, every increased or reduced value of certain groups of proteins is an indicator not only of the existence of some morbid process but also of the degree of its activity. 10

Various prior studies showed varying results with increased, decreased, and normal values of these. 11 These variations may be explained by the fact that the elevation of immunoglobulins may be due to the tumor cell antigens often elicit the production of specific serum antibodies. These antibodies can play a protective role in eliminating the tumor through several mechanisms. In some cases, the antibody can activate the complement system leading to assembly of the membrane attack complex, pore formation, and complement – mediated lysis. Antibodies bound to tumor cells may also facilitate the influx of inflammatory cells, especially neutrophils and macrophages. Antibodies bound to tumor cells may also facilitate antibody dependent cell mediated cytotoxicity (ADCC). Both macrophages and natural killer cells have receptors for the FC region of certain antibody classes. The antibody thus serves to bring these nonspecific immune cells into contact with the tumor.

Some patients who do not show any elevation and their immunoblobulins within the normal value may suggest a disturbance in the secretory immune system, and immunologic defect. 12

Dawood and Hasan found that the levels of serum IgG and IgA in patients with oral cancers were significantly increased when compared with controls. 13 Taneja et al in their study found serum levels of IgG and IgM were increased in oral submucous fibrosis cases. 14 The present study also compared serum IgG and serum IgA levels in oral precancers, oral cancers with that of controls which showed increased levels of IgG and IgA in precancer and oral cancer patients, which was statistically significant that highlights the role of active immune phenomena and accelerated body defense.

CIC are the regulator for both cellular and humoral response by their capacity to interact with antigen receptors. 15 Immune complexes are removed from the bloodstream by macrophages in the spleen and Kupffer cells in the liver under normal conditions. In some circumstances, however, immune complexes continue to circulate. 16

Immune complexes may themselves cause disease when they are deposited in organs, and this deposition is a prominent feature of several autoimmune diseases. 17 This occurs when the host's antibody production, relative to the amount of antigens, is inadequate for prompt elimination of antigen. 18

In some neoplasia, the quantity of CIC appears to be related to the tumor burden. 19 While in other, CIC does not appear to be related to prognosis. 20 This is mainly due to the difference that some tumors are poorly immunogenic, while in some antigens may be shed intermittently. This indicate that CIC levels cannot be used alone to determine the severity of the disease. Even though more of tumor antigens are shed into the system as the severity of the diseases increases, only a part of it binds to the antibody to form CIC. It has been observed that there is a fall in the circulating antibody levels in the course of rapid growth due to absorption of tumor antigens by tumor cell membranes. 21

Khanna and Karjodkar in their study found increased levels of CIC in oral cancer, followed by precancer and controls. 2 This is in contradiction to the present study where elevated levels of CIC were found in precancers compared with cancers and controls.

Jane et al assessed the serum CIC levels in oral precancers and different grades of OSCC and found the mean levels of CIC were increased in OSMF when compared with oral leukoplakia, and it was also seen to be elevated in different stages of oral cancer group. 1 In contrast to the present study where elevated levels of CIC were found in precancer group compared with cancer group. These variations may be due to the role of humoral immunity in pathogenesis of oral precancer when compared with oral cancer which has a multifactorial etiology.

## Conclusion

The rise in immunoglobulins and CIC may be nonspecific. Further studies should be performed with increased sample size and accompanied by studies on cell-mediated immunity should also be assessed in further studies to arrive at a specific conclusion.
